# Adherent Pericardium

**Published:** 1903-03-28

**Authors:** 


### ADHERENT PERICARDIUM.

In a space of 16 years there occurred among the in-patients of St. Mary's Free Hospital for Children, New York, 18 cases of adherent pericardium. That is to say, one case in every 300 was of this nature, admission being limited to children between the ages of two and 14 years. Basing his observations on these 18 cases, together with five others which he has met with in adults, Dr. G. M. Swift[1] discusses the affection, mainly in its clinical aspects. All the hospital cases ended fatally, and autopsies were performed in most of them. Usually it has been found that the outer surface of the pericardium is adherent to the chest wall and to the diaphragm, while the pericardial sac has become completely obliterated. The pericardium and heart muscle appear to be fused. The process seems to extend in some instances over a period of years, and is an active growing one. In long standing cases the appearance of the heart is one of enormous hypertrophy; probably there is less hypertrophy and more degeneration and dilatation than is sometimes taught. From the point of view of the clinician, it is certain that the condition prior to death has been rather that of a degenerated and dilated heart, and it is a fact, pointing to the same conclusion, that the administration of cardiac stimulants in this affection produces unfortunate effects. In many of the patients no valvular lesions have been present, the murmurs noticed before death being due to dilatation of the orifices. One lesion which Dr. Swift has observed is very great enlargement of the liver, greater, he thinks, than is met with in cases of dilated heart due to valvular disease. Two of his patients whom he was able to watch for periods of years showed an enlarged liver as the first indication of commencing failure. Rheumatic cases appear to follow a different course from those due to pneumococcus infection. The latter variety seems to occur earlier in life, and may be primary or secondary to a pleuro-pneumonia. The symptoms in all cases are those due to a dilated heart. Hearts with adherent pericardia are unusual in the marked irregularity and turbulency of their action. In three adult cases attacks of dyspnœa and apprehension of death were marked symptoms during the last months of life. Physical examination shows a bulging of the left chest, a large area of diffused impulse instead of a localised apex beat, and in some cases a recession of the intercostal spaces during systole. Percussion dulness is, of course, present over a larger area than is normal. On auscultation double murmurs are audible at base and apex. With regard to prognosis Dr. Swift notes that every one of the hospital cases had a fatal termination, but the private cases have been more successful, and he thinks a plentiful supply of nitrogenous food is essential for the patient's well-doing, while it is equally important to arrest the constant rheumatic poisoning.

[1] New York Medical News, Feb. 28.

